# Vascular and skeletal muscle oxidative capacity responses to continuous double and single leg cycling

**DOI:** 10.1113/EP093377

**Published:** 2026-02-15

**Authors:** Edward Z. Pelka, B. Ryan Davis, John McDaniel

**Affiliations:** ^1^ Exercise Science and Exercise Physiology Program Kent State University Kent Ohio USA; ^2^ Department of Kinesiology University of North Georgia Dahlonega USA; ^3^ Louis Stokes Cleveland Veterans Affairs Medical Center Cleveland Ohio USA

**Keywords:** aerobic exercise, blood flow, NIRS, vascular function

## Abstract

The purpose of this work was to determine endothelial, microvascular, skeletal muscle oxidative capacity (SMOC) and cardiorespiratory responses to an acute bout of continuous single leg cycling (SLC) and double leg cycling (DLC). Ten recreationally active men and women volunteered to participate in this investigation and reported to the laboratory on four separate occasions. Visits 1 and 2 consisted of a DLC and SLC ${\dot{V}}_{O_{2}\mathrm{peak}}$ test, while visits 3 and 4 were the experimental visits. Participants performed 30 min of continuous DLC and SLC at 60% of their DLC ${\dot{V}}_{O_{2}\mathrm{peak}}$. Before, 1 and 2 h post‐exercise, measures of vascular (i.e., flow mediated dilation (FMD), reactive hyperaemia, microvascular responsiveness) and SMOC were performed. SLC resulted in significantly greater limb specific power (83 ± 23 vs. 51 ± 12 W; *P *< 0.001) and carbohydrate oxidation (151 ± 40 vs. 126 ± 30 kcal; *P = *0.017) compared to DLC. There was a significant reduction in % FMD following SLC (baseline: 9.4 ± 3.2%; 1 h: 6.9 ± 3.4%; *P = *0.009), while there was no change following DLC. Both SLC and DLC resulted in a significant increase in SMOC (*P *< 0.001) and a significant decrease in microvascular responsiveness (*P *< 0.001). In conclusion, the reduction in FMD following SLC, likely brought on by greater peripheral and oxidative stress, which are key stimuli for long‐term positive adaptations, may be more beneficial at improving peripheral adaptations compared to DLC. This may be particularly advantageous for those with exercise intolerance, as SLC leads to greater peripheral stress for a similar central stress.

## Abstract (highlights)

- **What is the central question of this study?**
  What are the vascular responses following an acute bout of single and double leg cycling?
- **What is the main finding and its importance?**
  Flow mediated dilation is acutely reduced following single leg cycling, which could represent a transient step in the vascular remodelling process. Due to the lower central stress associated with single leg cycling, this exercise modality may be more beneficial for those with exercise intolerance and peripheral vascular dysfunction compared to traditional exercise modalities.

## 1 INTRODUCTION

Exercise intensity during large muscle mass aerobic exercise is limited by central circulation in healthy populations (Bassett & Howley, 2000). Specifically, as intensity increases cardiac output becomes unable to maintain adequate blood flow to the active muscles while maintaining appropriate mean arterial pressure. However, by performing small muscle mass exercise such as single leg cycling (SLC), cardiac output is no longer a limitation and skeletal muscle perfusion will exceed that from more traditional large muscle mass activity (Andersen & Saltin, 1985). When compared to double leg cycling (DLC), SLC allows one to cycle at a 10–21% greater limb specific power (Gordon et al., 2018; Iannetta et al., 2019) resulting in 30–90% greater limb specific blood flow (Burns et al., 2014; LaScola et al., 2020). The increase in limb specific workload, and subsequent peripheral stress, from SLC may result in greater positive adaptations for athletes (Abbiss et al., 2011) and those with central limitations such as chronic obstructive pulmonary disease (Dolmage & Goldstein, 2006). For example, the greater reliance on carbohydrate oxidation (LaScola et al., 2020) during SLC at similar power outputs compared to DLC may be beneficial for those with metabolic disease. Furthermore, lower ventilation and heart rate and greater overall exercise tolerance has been reported in those with chronic obstructive pulmonary disease (Dolmage & Goldstein, 2006) and idiopathic pulmonary fibrosis (Dolmage et al., 2020) during SLC, compared to DLC. However, acute vascular and mitochondrial function responses following an acute bout of SLC are unknown.

It is well established that vascular function (Bond et al., 2015; Kapilevich et al., 2020; Weston et al., 2022) and various biomarkers of mitochondrial function (Cochran et al., 2014; Kras et al., 2019; Little et al., 2011, 2010) are altered following an acute bout of aerobic exercise. Yet, these responses typically differ between moderate continuous and high intensity interval training. Previous reports indicate flow mediated dilation (FMD) is reduced immediately after high intensity interval training but returns back to or above baseline values 1 and 2 h post‐exercise (Bond et al., 2015; Weston et al., 2022). However, there is no change in FMD following moderate continuous exercise (Bond et al., 2015; Weston et al., 2022). These acute reductions in FMD following exercise appear to be due to the elevated oxidative stress following high intensity exercise (Dawson et al., 2013). While the transient reductions in FMD may appear to be unfavourable, limited evidence suggests the transient reduction in FMD post‐exercise represent an initial step in the structural remodelling process to the vascular system (Dawson et al., 2013; Gresele et al., 2007; Suvorava & Kojda, 2007). This idea reflects the common concept of hormesis, where repeated exposure to acute stimulus elicits improvement in function over time. Initial adaptations result in functional improvements in FMD due to an increase in endothelial nitric oxide synthase, followed by a subsequent decrement in FMD as conduit artery diameter increases (Green et al., 2017; Tinken et al., 2008). Therefore, the greater peripheral stress and fatigue associated with small muscle mass exercise (Rossman et al., 2014; Zhang et al., 2021) may lead to a significant reduction in FMD post‐exercise, and subsequent beneficial structural changes within the peripheral vasculature, compared to double leg cycling. Additionally, various mitochondrial biomarkers such as citrate synthase activity (Kras et al., 2019; Little et al., 2011) and peroxisome proliferator‐activated receptor γ coactivator 1‐α (PGC‐1α) content (Little et al., 2011, 2010) increase following high intensity interval and moderate continuous training, stimulating mitochondrial capacity and biogenesis. Collectively, this suggests exercise results in several acute changes to various biological systems, the magnitude of which may differ between exercise intensity.

Despite exercising at a similar whole‐body metabolic rate (i.e., ${\dot{V}}_{O_{2}}$) during SLC and DLC, the muscle specific intensity of SLC is much greater. To our knowledge there are no investigations that have analysed skeletal muscle oxidative capacity (SMOC), endothelial or microvascular function following an acute bout of SLC. Understanding the acute responses to these systems will help us understand the potential adaptations to SLC. By controlling ${\dot{V}}_{O_{2}}$, we aimed to isolate the effect of central versus peripheral stress on vascular function. Thus, the primary objective of this investigation is to compare an acute bout of DLC and SLC, matched for ${\dot{V}}_{O_{2}}$, on substrate oxidation, vascular function and SMOC. Based on previous findings we hypothesized for the same ${\dot{V}}_{O_{2}}$ across SLC and DLC, absolute workload (i.e., power) would be similar, while carbohydrate oxidation will be greater during SLC, compared to DLC. Due to the increase in peripheral stress from SLC, we hypothesize SLC will result in significant acute reductions in vascular function while DLC will not. Additionally, SMOC will increase following exercise in both conditions, similar to other mitochondrial biomarkers.

## 2 METHODS

### 2.1 Ethical approval

All participants were informed of the study design, risks and benefits prior to participation. All individuals who agreed to participate in the study completed a written informed consent. This protocol was approved and in accordance with the University's Institutional Review Board (IRB no. 1062) and adhered to the latest version of the *Declaration of Helsinki*, except for database registration.

### 2.2 Participants

A prior estimation of sample size was conducted using G*Power (G*Power, Heinrich‐Heine‐University, Dusseldorf, Germany). Effect size *(f*) was estimated from a previous investigation analysing FMD responses following moderate‐continuous and high intensity interval cycling (Johnson et al., 2012), which resulted in an estimated effect size of *f = *0.67. To determine sample size estimates, a repeated measures ANOVA, within‐factors interactions was utilized with *f = *0.67; α = 0.05; power = 0.80; two conditions (DLC, SLC) by three measures (pre, 1 and 2 h post‐exercise), which resulted in an estimated sample size of six. Therefore, to ensure statistical power was met, 10 recreationally active men (*n *= 6) and women (*n *= 4) were recruited and volunteered to participate in this investigation (173.2 ± 8.8 cm; 71.4 ± 12.3 kg; 25 ± 3 years; 15.3 ± 10.3% fat; 0.5 ± 0.2 cm adipose tissue thickness at NIRS site).

### 2.3 Experimental protocol

**FIGURE 1:**
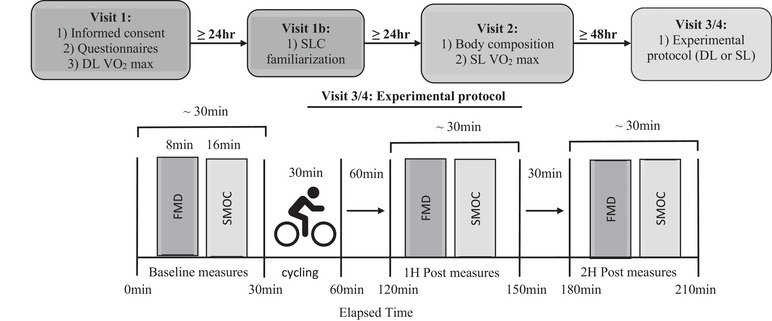
Experimental design. Participants (*n* = 10) reported to the laboratory on four separate visits for the investigation. Visits 3 and 4 were similar in design and consisted of the experimental protocol. FMD, flow mediated dilation; SMOC, skeletal muscle oxidative capacity.

Participants visited the laboratory on four separate days, with at least 48 h between each visit (Figure 1). During the initial visit, adipose tissue thickness on the thigh was assessed on the dominant leg via Doppler ultrasound (leg dominance assessed via Waterloo Footedness Questionnaire). Participants then completed a double leg ${\dot{V}}_{O_{2}\mathrm{peak}}$ test on a Velotron cycle ergometer (Racer Mate, Seattle, WA, USA) utilizing a Parvo Medics metabolic cart (Parvo Medics, Salt Lake City, UT, USA). Finally, participants performed a 5 min familiarization session of SLC. On visit 2, participants completed a three‐site skinfold assessment (males: chest, abdomen and thigh; females: triceps, suprailiac, and thigh) and a single leg ${\dot{V}}_{O_{2}\mathrm{peak}}$ test with their dominant leg. Visits 3 and 4 consisted of the experimental procedures, in which participants performed 30 min of either DLC or SLC, at 60% of their DLC ${\dot{V}}_{O_{2}\mathrm{peak}}$. Before exercise, 1 and 2 h post‐exercise, various measures of vascular function and SMOC were conducted on the dominant leg.

### 2.4 Cardiorespiratory fitness

A double and single leg cycling maximal exercise test was conducted on a Velotron cycle ergometer during visits 1 and 2, respectively. For DLC, participants cycled at 50 W for 3 min, followed by an increase in 1 W every 2 s until volitional fatigue, or when revolutions per minute dropped below 60 (MacInnis et al., 2017). During SLC, participants pedalled at 25 W for 3 min, followed by an increase of 1 W every 4 s (MacInnis et al., 2017). A 10‐kg counterweight was attached to the unoccupied crank arm to better replicate the biomechanics of DLC (Elmer et al., 2016). Gas exchange was continuously monitored via a Parvo Medics metabolic cart during both protocols. ${\dot{V}}_{O_{2}\mathrm{peak}}$ was determined as the highest ${\dot{V}}_{O_{2}}$ using a 30‐s rolling average (MacInnis et al., 2017). ${\dot{V}}_{O_{2}\mathrm{peak}}$ was further supported when individuals also had a respiratory exchange ratio (RER) >1.10 and/or heart rate was within 10 beats per minute (bpm) of age predicted max (which may not occur during SLC).

### 2.5 Single and double leg cycling

During visits 3 and 4 participants completed a 30 min bout of either SLC or DLC at 60% of their double leg cycling ${\dot{V}}_{O_{2}\mathrm{peak}}$. Throughout the protocol, power was adjusted in 5 W increments or decrements to maintain desired ${\dot{V}}_{O_{2}}$. Prior to the start of the cycling protocol, a PortaMon (Artinis Medical systems, Elst, Netherlands) near‐infrared spectroscopy (NIRS) device was placed on the vastus lateralis. During cycling, at minutes 10, 20 and 30, participants were provided with a 1 min break. During this break, a 10 cm Hokanson rapid inflating blood pressure cuff (E20 rapid cuff inflator, Hokanson, Bellevue, WA, USA) was placed around the thigh superior to the NIRS device and inflated to 250–300 mmHg for 10 s to prevent blood flow into the limb. This occlusion allowed for the assessment of skeletal muscle oxygen consumption ($m{\dot{V}}_{O_{2}}$) calculated as the rate change of oxyhaemoglobin during the cuff occlusion (Ryan et al., 2013a). Similar to the SLC ${\dot{V}}_{O_{2}}$ test, during SLC trial participants cycled with their dominant leg and a 10‐kg counterweight was attached to the unoccupied crank arm.

### 2.6 Substrate utilization

Following both SLC and DLC experimental protocols, all metabolic data were exported in 20‐s averages to a custom‐built Microsoft Excel template. For carbohydrate and fat oxidation, stoichiometry equations were used based off of moderate‐to‐high intensity exercise (Jeukendrup & Wallis, 2005). Specifically, for carbohydrate, oxidation rate (g/min) = (4.210 × ${\dot{V}}_{CO_{2}}$) − (2.962 × ${\dot{V}}_{O_{2}}$), where 1 g of carbohydrate is equivalent to 4.07 kcal. For fat, oxidation rate (g/min) = (1.695 × ${\dot{V}}_{O_{2}}$) − (1.701 × ${\dot{V}}_{CO_{2}}$), where 1 g of fat is equivalent to 9.75 kcal. Substrate oxidation was determined and quantified once steady state exercise was achieved (defined as minutes 5–30). If RER was >1.0, which sometimes occurred during SLC, ${\dot{V}}_{CO_{2}}$ was matched to ${\dot{V}}_{O_{2}}$ values (i.e., RER = 1.0) to avoid overestimation of CHO oxidation due to non‐metabolic CO_2_ production. This correction was applied to 153 of 1800 data points (8.5%). Such adjustment likely resulted in underestimation of CHO oxidation for these participants.

### 2.7 Vascular function

Vascular function was assessed on the superficial femoral artery via a GE Logiq 7 Doppler/ultrasound utilizing two separate techniques: FMD and reactive hyperaemia. A standard FMD protocol (Harris et al., 2010; Thijssen et al., 2011) was conducted on the dominant leg while following guidelines used for assessing FMD responses to the acute exercise model (Padilla et al., 2007). Briefly, participants laid supine with a Hokanson rapid inflating blood pressure cuff wrapped around the thigh and an ultrasound probe placed over the superficial femoral artery proximal to the cuff. Resting arterial diameter and blood velocity of the superficial femoral artery was recorded for 60 s and then the cuff was inflated to approximately 250–300 mmHg for 5 min to perform an arterial occlusion. After 5 min, the cuff was deflated and arterial diameter and blood velocity were recorded for 2 min. All ultrasound data were transferred to a computer where arterial diameter was analysed via Vascular Research Tools edge detection software (Medical Imaging Applications LLC, Coralville, IA, USA). Blood flow (mL/min) was analysed and calculated as [(π × (diam/2)^2^ × (blood velocity) × 60], while shear was calculated as [(blood velocity*8)/diameter)]. Cumulative shear rate area under the curve (SRauc) was calculated until the point of peak dilation. Percentage FMD was determined as the increase in peak arterial diameter compared to resting diameter and expressed relative to cumulative shear to the point of peak dilation (Harris et al., 2010). Reactive hyperaemia following the cuff release was calculated as peak blood flow response, as well as total blood flow response throughout the 2 min.

### 2.8 Skeletal muscle oxidative capacity

Participants were seated in a chair with a PortaMon NIRS device placed longitudinally on the vastus lateralis and a Hokanson rapid inflating blood pressure cuff superior to it. To prevent ambient light from reaching the sensor, black Coban was wrapped around the PortaMon. The PortaMon measures oxygenated haemoglobin (O_2_HB), deoxygenated haemoglobin (HHB), and total haemoglobin (tHB) continuously via 760 and 850 nm wavelengths and has an optode distance of 35 mm.

After 5 min of seated rest, a 10 s arterial occlusion was performed by inflating the Hokanson to 250–300 mmHg (Ryan et al., 2013a) to measure $m{\dot{V}}_{O_{2}}$ as the rate decline in oxygenated haemoglobin. Three baseline measures of $m{\dot{V}}_{O_{2}}$ were performed, with 50 s of rest between each. Participants then cycled for 5 min at a workload corresponding to approximately 40% of their maximal power performed during their DLC ${\dot{V}}_{O_{2}\mathrm{peak}}$ test. Exactly 15 s after the cessation of cycling a series of 12 repeated arterial occlusions were performed to determine recovery of $m{\dot{V}}_{O_{2}}$. Occlusions 1–5 were 5 s on, 5 s off, occlusions 5–10 were 5 s on, 10 s off, and occlusions 11–12 were 10 s on, 20 s off (Ryan et al., 2013a). Following these occlusions, a 5 min arterial occlusion was conducted in order to create a physiological calibration. This procedure provides a range from maximal to minimal physiological O_2_HB concentration, such that individual $m{\dot{V}}_{O_{2}}$ slopes can be normalized allowing for better comparisons across individuals. In addition to this normalization, another variable that comes out of this protocol is half‐time to peak hyperaemia, which assess microvascular responsiveness, and was calculated as the amount of time it took to reach 50% of peak O_2_HB following the release of the 5 min cuff occlusion (Willingham et al., 2016).

All PortaMon data were then exported to a custom built Microsoft Excel template and corrected for blood volume shifts (Beever et al., 2020) for each channel, and $m{\dot{V}}_{O_{2}}$ was calculated as the slope of O_2_HB during each occlusion. Each slope was then plotted on a mono‐exponential curve (Ryan et al., 2013b): [*y*(*t*) = End − Δ × e^(−^ *^kt^* ^)^] where *y*(*t*) is relative $m{\dot{V}}_{O_{2}}$ during arterial occlusions (O_2_Hb slopes) at the time *t*; End is $m{\dot{V}}_{O_{2}}$ immediately following exercise; Δ is difference between the resting $m{\dot{V}}_{O_{2}}$ and End; and *k* is a rate constant representing the SMOC (Ryan et al., 2013a).

### 2.9 Statistical analysis

All data were analysed via SPSS Statistics software version 29.0 (IBM Corp., Armonk, NY, USA), with α set at 0.05. ${\dot{V}}_{O_{2}\mathrm{peak}}$, max heart rate and max power were compared between DLC and SLC maximal exercise tests. In addition, during the experimental trials, ${\dot{V}}_{O_{2}}$, HR, CHO, fat and kcal were compared across the two conditions via a paired samples Student's *t*‐test. All Doppler (resting diameter, resting blood flow, %FMD, %FMD/shear and reactive hyperaemia) and NIRS (SMOC and half‐time to peak hyperaemia) data were analysed via a two‐condition (DLC, SLC) by three time points (baseline, 1 and 2 h post) repeated measures ANOVA. If a significant interaction occurred, paired samples *t*‐tests with a Benjamini–Hochberg *post hoc* analysis was conducted to determine where the differences occurred. Pearson's product‐moment correlation analyses were conducted between average limb‐specific power during exercise and the change (Δ) in each %FMD, half‐time to peak hyperaemia and skeletal muscle oxidative capacity from baseline to 1 h post‐exercise.

## 3 RESULTS

### 3.1 Physiological responses during exercise

${\dot{V}}_{O_{2}\mathrm{peak}}$ (39.6 ± 6.8 vs. 32.4 ± 5.6 mL/kg/min; *t *= 4.797; *P = *0.005) and heart rate max (181 ± 11 vs. 173 ± 15 bpm; *t *= 2.791; *P = *0.027) were significantly greater during double leg max testing, compared to single leg max testing (Table 1). However, limb specific power was significantly greater during single leg max testing, compared to double leg max testing (150 ± 32 vs. 134 ± 30 W; *t = *6.488; *P = *0.003). During 30 min SLC and DLC trials, power was adjusted to control for ${\dot{V}}_{O_{2}}$. Thus, there was no condition by time interaction (*f = *1.133, *P = *0.344), main effect of condition (*f = *0.001, *P = *0.979), or main effect of time (*f = *3.051, *P = *0.072) for $m{\dot{V}}_{O_{2}}$ (Table 2). However, the similarity in ${\dot{V}}_{O_{2}}$ occurred at a higher limb specific power, average heart rate, carbohydrate utilization and a significantly lower fat utilization for SLC compared to DLC (Table 1).

**TABLE 1:** Physiological responses to 30 min of double and single leg cycling at 60% of double leg ${\dot{V}}_{O_{2}\max}$.

Variable	Double leg cycling	Single leg cycling	*t*	*P*
Average ${\dot{V}}_{O_{2}}$ (mL/kg/min)	22.0 ± 3.2	21.5 ± 3.3	1.457	0.201
Average heart rate (bpm)	138 ± 13	148 ± 10	4.317	**0.005**
Average limb specific power (W)	51 ± 12	83 ± 23	8.321	**0.001**
Carbohydrate kcal	126 ± 30	151 ± 40	2.908	**0.017**
Fat kcal	73 ± 34	44 ± 19	3.768	**0.004**
Total kcal	199 ± 47	195 ± 47	1.042	0.324

*Note*: All data expressed as mean ± SD. Sample size: *n* = 10. *P*‐values were calculated using paired samples *t*‐tests. Abbreviations: bpm, beats per minute.

**TABLE 2:** Measures of $m{\dot{V}}_{O_{2}}$ between double and single leg cycling at 60% of double leg ${\dot{V}}_{O_{2}\max}$.

Variable	10 min	20 min	30 min	*P*‐value
Double leg (%O_2_Hb change/s)	5.04 ± 0.99	4.90 ± 1.19	5.98 ± 2.16	Interaction = 0.334 Condition = 0.979 Time = 0.072
Single leg (%O_2_Hb change/s)	4.22 ± 1.22	5.47 ± 1.49	6.25 ± 3.32	

*Note*: All data expressed as means ± SD. Sample size: *n* = 10. Abbreviations: DLC, double leg cycling; O_2_Hb, oxygenated haemoglobin; SLC, single leg cycling.

### 3.2 Vascular function

There was no condition by time interaction (*f = *1.160, *P = *0.336), main effect of condition (*f = *0.700, *P = *0.158) or main effect of time (*f = *1.271, *P = *0.305) for resting superficial femoral arterial diameter. Additionally, there was no condition by time interaction (*f = *1.166, *P = *0.334), main effect of condition (*f = *0.534, *P = *0.483) or main effect of time (*f = *1.361, *P = *0.282) for resting blood flow (Table 3).

**TABLE 3:** Resting superficial femoral arterial diameter and blood flow pre‐exercise, 1 h and 2 h post‐exercise.

Variable		Pre‐exercise	1 h Post	2 h Post	*P*
Resting arterial diameter (mm)	DLC	6.13 ± 0.80	6.11 ± 0.80	6.13 ± 0.80	Interaction = 0.336 Condition = 0.158 Time = 0.305
	SLC	6.12 ± 0.81	6.12 ± 0.80	6.12 ± 0.80	
Resting blood flow (mL/min)	DLC	100.4 ± 44.5	95.9 ± 37.1	92.0 ± 31.3	Interaction = 0.334 Condition = 0.483 Time = 0.282
	SLC	107.3 ± 75.8	78.8 ± 22.5	86.3 ± 35.6	

*Note*: All data expressed as means ± SD. Sample size: *n* = 10. Abbreviations: DLC, double leg cycling; SLC, single leg cycling.

**FIGURE 2:**
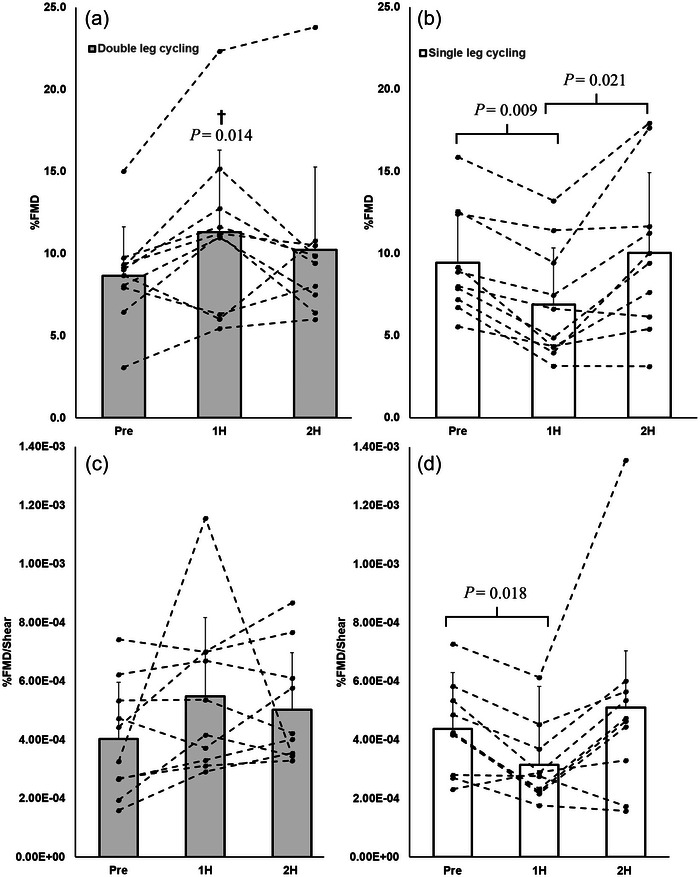
Flow‐mediated dilation responses to 30 min of double and single leg cycling. Grey bars represent double leg cycling, while white bars represent single leg cycling. Sample size: *n* = 10. Data presented as means ± SD with individuals data points. A 2 × 3 repeated measures ANOVA with a Benjamini–Hochberg *post hoc* analysis was performed with α set at 0.05. †Significantly different between single leg cycling. FMD, flow mediated dilation; SMOC, skeletal muscle oxidative capacity.

Analysis indicated a significant condition by time interaction (*f = *16.458, *P *< 0.001), but no main effect of condition (*f = *1.424, *P = *0.263) or main effect of time (*f = *1.464, *P = *0.258) on %FMD. *Post hoc* analysis revealed %FMD significantly decreased from 9.4 ± 3.2% to 6.9 ± 3.4% between SLC pre and SLC 1 h post (*t = *6.381, *P = *0.009), %FMD significant increased from 6.9 ± 3.4% to 10 ± 4.9% between SLC 1 h post and SLC 2 h post (*t = *3.442, *P = *0.021), and %FMD was 4.4% greater for DLC 1 h post compared to SLC 1 h post (*t = *3.939, *P = *0.014) (Figure 2a). When %FMD was made relative to shear, there was a significant condition by time interaction (*f = *4.368, *P = *0.028), but no main effect of condition (*f = *1.307, *P = *0.282) or main effect of time (*f = *1.852, *P = *0.186). *Post hoc* analysis revealed %FMD/shear significantly decreased from 4.38 × 10^−4^ ± 1.54 × 10^−4^ to 3.14 × 10^−4^ ± 1.32 × 10^−4^ between SLC Pre and SLC 1 h post (*t = *4.179, *P = *0.018) (Figure 2b). For cumulative SRauc, there was no condition by time interaction (*f *= 0.342, *P *= 0.715), main effect of condition (*f *= 0.111, *P *= 0.747) or main effect of time (*f *= 1.406, *P *= 0.271). Additionally, there was no condition by time interaction (*f *= 0.271, *P *= 0.766), main effect of condition (*f *= 0.130, *P *= 0.726) or main effect of time (*f *= 0.218, *P *= 0.806) for time to peak dilation (Table 4).

**TABLE 4:** Reactive hyperaemia and skeletal muscle oxidative capacity responses to 30 min of double and single leg cycling.

Variable		Pre‐exercise	1 h Post	2 h Post	*P*
Shear rate AUC (AU)	DLC	23976.9 ± 8777.0	22076.4 ± 7719.5	20422.0 ± 4329.7	Interaction = 0.715 Condition = 0.747 Time = 0.271
	SLC	22870.2 ± 7461.3	21349.7 ± 3783.4	21425.1 ± 6411.7	
Time to peak dilation (s)	DLC	62 ± 19	53 ± 24	59 ± 33	Interaction = 0.766 Condition = 0.726 Time = 0.806
	SLC	59 ± 21	59 ± 31	63 ± 27	
Reactive hyperaemia peak flow (mL/min)	DLC	1265.4 ± 423.9	1417.2 ± 516.0	1396.7 ± 492.5	Interaction = 0.471 Condition = 0.730 Time = 0.577
	SLC	1330.2 ± 476.2	1351.5 ± 483.5	1346.2 ± 513.0	
Reactive hyperaemia AUC (mL)	DLC	543.7 ± 257.4	547.3 ± 247.5	489.5 ± 152.3	Interaction = 0.952 Condition = 0.746 Time = 0.242
	SLC	534.8 ± 205.4	527.5 ± 202.0	493.2 ± 202.4	
*K*‐constant (min^−1^)	DLC	1.00 ± 0.23	1.32 ± 0.36	1.14 ± 0.28	Interaction = 0.376 Condition = 0.229 **Time < 0.001**
	SLC	1.00 ± 0.27	1.36 ± 0.48	1.34 ± 0.21	
Half‐time to peak O_2_Hb hyperaemia (s)	DLC	13.1 ± 3.0	11.7 ± 2.3	10.2 ± 2.4	Interaction = 0.268 Condition = 0.851 **Time < 0.001**
	SLC	14.3 ± 2.9	11.2 ± 1.9	9.9 ± 1.6	

*Note*: All data expressed as means ± SD. Sample size: *n* = 10. Abbreviations: AU, arbitrary units; AUC, area under the curve; DLC, double leg cycling; SLC, single leg cycling.

There was no condition by time interaction (*f = *0.786, *P = *0.471), main effect of condition (*f = *0.127, *P = *0.730) or main effect of time (*f = *0.567, *P = *0.577) for reactive hyperaemia peak blood flow, and there was no condition by time interaction (*f = *0.050, *P = *0.952), main effect of condition (*f = *0.112, *P = *0.746) or main effect of time (*f = *1.536, *P = *0.242) for reactive hyperaemia area under the curve (AUC). Additionally, there was no condition by time interaction (*f = *1.976, *P = *0.268) or main effect of condition (*f = *0.037, *P = *0.851); however, there was a significant main effect of time (*f = *23.225, *P *< 0.001) for microvascular responsiveness, in which microvascular responsiveness decreased over time. (Table 4).

### 3.3 Skeletal muscle oxidative capacity

There was no condition by time interaction (*f = *1.032, *P = *0.376) or main effect of condition (*f = *1.668, *P = *0.229); however, there was a significant main effect of time (*f = *10.646, *P *< 0.001) for SMOC (*K*‐constant), in which the *K*‐constants increased over time (Table 4).

### 3.4 Correlations

When both exercise conditions were collapsed into one group, limb specific power resulted in a significant moderate negative correlations with Δ (1 h minus baseline) %FMD (*r* = −0.586, *P* = 0.007), significant moderate positive correlations with Δ *K*‐constant (*r* = 0.448, *P* = 0.047) and no correlation with Δ half‐time to peak hyperaemia (*r* = −0.095, *P* = 0.690).

## 4 DISCUSSION

To our knowledge, this is the first investigation to determine changes in vascular function and SMOC responses to an acute bout of SLC. Our data suggests SLC at similar ${\dot{V}}_{O_{2}}$ compared to DLC results in significantly greater carbohydrate oxidation, heart rate and limb specific power. Post‐exercise, SLC results in a significant reduction in FMD, while FMD is not altered following DLC. Microvascular function is not altered following SLC and DLC when assessed via reactive hyperaemia; however, it is improved when assessed via half‐time to peak hyperaemia. Additionally, SLC and DLC both result in similar increases in SMOC. Overall, SLC provides a robust stimulus that results in acute decrements to %FMD and increases in SMOC, which may lead to greater increases in arterial diameter and oxidative capacity, compared to DLC.

### 4.1 Physiological responses during exercise

During maximal exercise testing, SLC resulted in a significantly greater limb specific power for a lower central stress (i.e., ${\dot{V}}_{O_{2}}$ and HR), compared to DLC, which is in agreement with previously published literature (Iannetta et al., 2019). This suggests SLC can be effective at enhancing peripheral muscular work, while minimizing central cardiovascular strain. Thirty minutes of DLC and SLC at matched ${\dot{V}}_{O_{2}}$ was accomplished at greater limb specific power, heart rate and carbohydrate oxidation during SLC compared to DLC (Table 1). These data are in partial agreement with previous investigations that compared cardiorespiratory responses to DLC and SLC (Burns et al., 2014; LaScola et al., 2020). When cycling at the same absolute workload, ${\dot{V}}_{O_{2}}$ and heart rate were similar at low‐to‐moderate exercise intensities, while RER was greater in SLC compared to DLC in young healthy individuals (Burns et al., 2014) and older men (LaScola et al., 2020). However, heart rate was greater during SLC at higher exercise intensities (i.e., 120 W) (Burns et al., 2014). Our data agree that SLC results in 62% greater limb specific power and 20% greater carbohydrate oxidation for similar systemic ${\dot{V}}_{O_{2}}$ values compared to DLC. This suggests SLC may be a useful exercise modality for those with insulin resistance and exercise intolerance. However, our data do not agree that heart rate is similar between DLC and SLC, which may be explained due to differences in exercise protocol. The aforementioned investigations completed DLC and SLC across three 4 min stages, separated by a recovery period, ranging from 25 to 75 W (LaScola et al., 2020) and 40 to 120 W (Burns et al., 2014). Therefore, the exercise intensity and/or duration may have been too low to distinguish differences in heart rate, especially since heart rate was significantly greater during DLC at 120 W (Burns et al., 2014). This increase in heart rate during SLC may be explained from a greater involvement of the exercise pressor reflex (Stavres et al., 2020) due to the greater limb specific power and peripheral stress in the single active limb during SLC.

Throughout exercise, $m{\dot{V}}_{O_{2}}$ was not significantly different between DLC and SLC (Table 2). Previous investigations have reported SLC results in a 30–90% increase in limb specific blood flow compared to DLC (Burns et al., 2014; LaScola et al., 2020), which likely explains the ability to perform greater limb specific work during SLC. However, our data suggests despite this increase in limb specific blood flow, $m{\dot{V}}_{O_{2}}$ of the vastus lateralis did not change. These results may be explained due to the measurement technique. Changes in superficial femoral blood flow account for changes in blood flow for the entire limb, while NIRS derived measurements of $m{\dot{V}}_{O_{2}}$ only account for changes of haemoglobin concentrations at the site of the NIRS device, which has a penetration depth of about 1.75 cm. Placing the NIRS at the site of the vastus lateralis does not account for changes in $m{\dot{V}}_{O_{2}}$ deeper into the vastus lateralis or in other active muscle groups. Previous studies using time‐resolved NIRS capable of assessing both deep and superficial muscle layers support the idea that localized measurements (i.e., superficial single site measures) may not fully capture whole‐muscle oxygen consumption (Koga et al., 2019; Okushima et al., 2015). Therefore, other muscle groups (i.e., stabilizing muscles) may become more active during SLC (Bini et al., 2015), thus increasing oxygen consumption.

### 4.2 Vascular function

In the present study, vascular function was assessed via FMD, reactive hyperaemia and microvascular responsiveness. To our knowledge, this is the first investigation to measure microvascular responsiveness pre‐ to post‐exercise. Regardless of exercise modality, reactive hyperaemia (i.e., peak blood flow and AUC) also did not change post‐exercise for either DLC or SLC (Table 4). Interestingly, microvascular responsiveness did improve following SLC and DLC. These data suggest acute aerobic exercise at moderate‐to‐high intensity may influence certain aspects of microvascular function. However, these results do not agree with a previous investigation that reported an increase in reactive hyperaemia following an acute bout of moderate continuous and high intensity interval training (Bond et al., 2015). Specifically, these investigators reported that both exercise intensities resulted in a significant increase in reactive hyperaemia immediately post, 1 and 2 h post‐exercise, but with a greater increase in the high intensity interval training group (Bond et al., 2015). A possible explanation for these differences could be the measurement location. The present investigation measured microvascular function within the exercising limb (i.e., downstream resistant vessels to the superficial femoral artery as assessed via reactive hyperaemia) in young healthy adults, while the previous investigation measured microvascular function within the non‐exercise limb (i.e., downstream resistant vessels to the brachial artery as assessed via reactive hyperaemia) in adolescences. Previously, it has been reported that microvascular function of the arm was not significantly correlated with microvascular function of the leg (Rossman et al., 2016). This suggests microvascular function may be responding to exercise differently between upper and lower limbs or differently due to local (within the active musculature) or systemic (outside the active musculature) effects. Thus, future investigations should assess changes in micro and macro vascular function for both upper and lower limbs following exercise.

Our data suggests SLC results in significant reductions in superficial femoral FMD 1 h post‐exercise, while DLC does not alter FMD (Figure 2). These data are in partial agreement with previous studies investigating brachial FMD following moderate (Bond et al., 2015; Weston et al., 2022) and high intensity exercise (Bailey et al., 2012; Bond et al., 2015; Harris et al., 2008; Rognmo et al., 2008; Weston et al., 2022). Following moderate intensity exercise, previous investigation have reported no change in brachial FMD (Bond et al., 2015; Weston et al., 2022), which agrees with our data that indicates there were no changes in superficial femoral FMD following continuous DLC. However, previous reports on vascular function following high intensity exercise have conflicting results. Specifically, previous reports indicate significant reductions in brachial FMD immediately post‐exercise (Bailey et al., 2012; Bond et al., 2015), 1 h post‐exercise in trained (Rognmo et al., 2008) and untrained individuals (Harris et al., 2008; Jurva et al., 2006), as well as an increase in brachial FMD 1, 2 and 3 h (Bond et al., 2015; Weston et al., 2022) post high intensity training. Our data suggests that the alterations is FMD following exercise are not dependent on whole body metabolic stress, as ${\dot{V}}_{O_{2}}$ was similar between DLC and SLC in our study. Rather the changes in FMD are more likely due to greater peripheral stress/fatigue as the limb specific power was significantly negatively correlated with %FMD.

The transient reduction in superficial FMD (1 h post) may have occurred due to a larger increase in oxidative stress following SLC. As exercise intensity increases, there is a larger increase in oxidative stress (Goto et al., 2003), which can lead to a reduction in nitric oxide bioavailability and reduce FMD (Dawson et al., 2013). Therefore, SLC may lead to a larger increase in oxidative stress compared to DLC, despite similar whole body ${\dot{V}}_{O_{2}}$, and transiently reducing FMD. Some markers of oxidative stress such as thiobarbituric acid‐reactive substances have been reported to peak 1 h post‐exhaustive exercise (Michailidis et al., 2007), which may explain the reduction of FMD 1 h post‐SLC. Additionally, previous reports indicate this acute reduction in FMD is followed by an acute heightened response that may occur 1–24 h post‐exercise, which is believed to occur due to an influx of antioxidants (Dawson et al., 2013). Although we did not see a heightened response in the current study, FMD did return to baseline at the 2 h mark.

### 4.3 Skeletal muscle oxidative capacity and half‐time to peak hyperaemia

Despite several reports that compare differences in SMOC across various populations such as healthy ageing (Fennell et al., 2023), COPD (Adami et al., 2017), heart failure (Southern et al., 2015) and spinal cord injuries (Erickson et al., 2013), to our knowledge, there are no reports on potential acute changes in SMOC and half‐time to peak hyperaemia responses following exercise. Our data suggest DLC and SLC resulted in significant improvements in SMOC; however, there were no differences between modalities (Table 4). The increase in SMOC may be explained, in part, due to a ‘priming effect’ and enhanced substrate provision, where prior heavy exercise could increase the activity of rate‐limiting oxidative enzymes (Goulding et al., 2023). Since SMOC is a component of mitochondrial and microvascular function, various mitochondrial biomarkers may have contributed to the increase in SMOC post‐exercise. Similar results have been previously reported in which an acute bout of moderate continuous and high intensity interval training resulted in similar significant increases in PGC‐1α mRNA expression 3 h post‐exercise (Cochran et al., 2014). Additionally, it has been reported that citrate synthase activity increases following an acute bout of moderate continuous (Kras et al., 2019) and high intensity interval training (Little et al., 2011). Therefore, our data support the aforementioned investigations in which acute exercise, regardless of intensity, improves various components of mitochondrial function/activity. Additionally, the improvement in SMOC may have resulted from an increase in skeletal muscle perfusion, which is noted from the increase in microvascular responsiveness (Table 4). Collectively, this suggests acute exercise can be utilized as a therapeutic modality to acutely improve these variables in various clinical populations.

### 4.4 Limitations

The present study is not without limitations that should be addressed. The sample size is relatively small and consists of young healthy individuals. Thus, these results may not apply to other population groups. Additionally, since post‐exercise measurements were only taken 1 and 2 h post‐exercise, the time course of the potential heighted FMD response post‐SLC is unknown. Another limitation of the present study is within NIRS technology. The penetration depth of the PortaMon is approximately 1.75 cm, which only accounts for a small portion of muscle tissue. Additionally, $m{\dot{V}}_{O_{2}}$ and SMOC were only performed on the vastus lateralis. Our data suggest $m{\dot{V}}_{O_{2}}$ was not significantly different between protocols (Table 2), despite the increase in limb specific power for SLC. Therefore, it is likely $m{\dot{V}}_{O_{2}}$ was greater in other muscle groups during SLC that we did not measure, due to small differences in SLC biomechanics (Elmer et al., 2016).

### 4.5 Conclusion

In conclusion, SLC results in an acute reduction in FMD (which may represent a transient step in the adaptation process) and increase in skeletal muscle oxidative capacity, which may in part be explained from the increase in peripheral work/stress. These responses may prove to be more beneficial compared to more traditional exercise modalities such as DLC. This suggests SLC can be utilized as a useful exercise modality for various clinical and athletic populations. Future research should examine vascular and mitochondrial adaptations to SLC and determine appropriate implementation training guidelines.

## AUTHOR CONTRIBUTIONS

Edward Z. Pelka, B. Ryan Davis, and John McDaniel contributed to the study conception and design. Edward Z. Pelka and B. Ryan Davis contributed to data collection. Edward Z. Pelka contributed to data analysis. Edward Z. Pelka and John McDaniel contributed to the original draft of the manuscript. Edward Z. Pelka, B. Ryan Davis, and John McDaniel contributed to the review and editing of the manuscript. All authors read and approved the final manuscript and agree to be accountable for all aspects of the work in ensuring that questions related to the accuracy or integrity of any part of the work are appropriately investigated and resolved. All persons designated as authors qualify for authorship, and those who qualify for authorship are listed.

## CONFLICT OF INTEREST

The authors declare no conflicts of interest.

## FUNDING INFORMATION

The authors declare no external funding support.

## Data Availability

Data are available upon request.
